# Deconstructing urban green justice and well-being: a multi-group structural equation modeling analysis based on the activity space perspective

**DOI:** 10.3389/fpubh.2025.1670454

**Published:** 2025-09-17

**Authors:** Shaobo Liu, Jialing Qi, Yi Yang, Wen Huo, Jiang Li, Yating Chang, You Peng

**Affiliations:** ^1^Department of Environmental Design, School of Architecture and Art, Central South University, Changsha, China; ^2^Human Settlements Research Center, Central South University, Changsha, Hunan, China; ^3^The Establishment of the Key Laboratory for High-density Habitat Ecology and Energy Conservation of Ministry of Education, TongJi University, Shanghai, China; ^4^Department of Architecture, College of Design and Engineering, National University of Singapore, Singapore, Singapore

**Keywords:** green justice, well-being, green space exposure, green space quality, activity space, multi-group structural equation modeling

## Abstract

**Introduction:**

Against the backdrop of a global shift toward greener cities, equitable access to urban green spaces has increasingly been recognized for its impact on residents’ well-being. However, most existing studies rely heavily on residential proximity, overlooking the role of individual activity space differences and their influence on perceived fairness and well-being.

**Methods:**

This study, grounded in environmental justice theory and the activity space perspective, constructs and tests a structural equation model integrating green space quality, green space exposure, perceived social benefits, perceived green justice, and subjective well-being. Based on 524 survey responses collected in Changsha, China, a multi-group structural equation modeling (Multi-Group SEM) approach was employed to compare path differences among near-, mid-, and far-distance activity space groups.

**Results and discussion:**

Results show that: (1) Urban green space quality significantly influences green space exposure and perceived social benefits, which in turn enhance residents’ perception of green justice and ultimately boost subjective well-being; (2) The pathways from “Green Space Quality → Green Space Exposure,” “Green Space Quality → Perceived Social Benefits,” and “Green Space Exposure → Perceived Green Justice” remain consistently significant across all activity groups, forming a stable core mechanism; (3) Green space exposure in the mid-distance group is more sensitive to economic conditions. By incorporating activity space segmentation, this study extends the micro-mechanism framework of environmental justice and subjective well-being. The findings provide empirical evidence for understanding the psychological impacts of green space equity on urban residents and offer theoretical support for precision-oriented green space planning and equity-focused policy interventions.

## 1 Introduction

With the rapid acceleration of global urbanization, urban park green spaces (UPGS) have become increasingly vital to sustainable urban development. According to United Nations projections, approximately 55% of the global population currently resides in urban areas, a figure expected to rise to 68% by 2050 (1). Urban areas are facing mounting ecological, environmental, and social pressures (2). In high-density urban environments, parks—one of the most publicly accessible components of urban green infrastructure—serve crucial ecological functions, such as mitigating the urban heat island effect (3), improving local microclimates (4), and reducing air and noise pollution (5). In addition, they provide significant social and health benefits, including promoting mental well-being (6), strengthening community cohesion (7), and enhancing social interaction (8).

However, as urban areas become more densely populated and land competition intensifies, the scarcity and uneven spatial distribution of UPGS are becoming increasingly problematic. Earlier research predominantly focused on the ecological functions of UPGS—such as mitigating heat islands (9, 10) and providing ecosystem services (11)—as well as health and well-being outcomes, including enhanced physical and mental health (6) and improved quality of life (12). In recent years, the emergence of environmental justice theory has shifted scholarly attention toward the spatial equity of UPGS. Some studies have revealed that complex socio-economic factors associated with urbanization contribute to disparities in green space’access across different locations and population groups. These disparities are often evaluated through socio-economic indicators such as age, education, poverty, income, and ethnicity (13–18). For instance, Jin Rui et al. investigated the impacts of green space inequality on spatial heterogeneity among vulnerable groups and its welfare consequences (14). Another study focusing on gender differences identified systemic disadvantages faced by women in accessing green space (19). At the same time, an increasing number of studies employ macro-level geospatial analyses and streetscape imagery to quantify green space equity. Common measures include spatial accessibility, green coverage, street-level greenness indices, usage opportunities, and per capita green space (13, 20–24). For example, Lu Shan et al. found that suburban parks significantly enhance the spatial accessibility of urban green space, thereby promoting environmental justice (25). Despite extensive efforts to evaluate spatial distributions of UPGS and related social inequities, empirical research remains limited regarding how subjective perceptions—shaped by household characteristics and social attributes—affect residents’ green space exposure (including visit frequency and travel time), and how these perceptions further influence perceived green space quality, social benefits, environmental justice, and overall well-being.

Furthermore, as urban spatial structures undergo increasing diversification, the traditional proximity-based perspective centered on residential location is revealing inherent limitations. A substantial body of existing research implicitly assumes spatial behavioral homogeneity among residents, primarily focusing on the accessibility of green spaces near their homes, while overlooking variations in spatial exposure during actual daily activities. However, residents exhibit significant differences in their daily activity spaces and mobility patterns, which in turn affect the frequency, type, and quality of green space contact across different groups (26–29). In recent years, the theory of activity space has introduced a dynamic and individualized perspective for analyzing environmental exposure, emphasizing individuals’ actual movement trajectories rather than static residential locations. This approach has been widely adopted in domains such as transportation, public health, and environmental behavior (30). In the context of urban green space equity, incorporating the activity space perspective enables a shift beyond the traditional static analytical framework, allowing for a more nuanced understanding of how variations in daily mobility influence green space access, perceived justice, and subjective well-being. This dynamic lens provides a theoretical basis for developing more targeted and equitable urban policies.

Building on this foundation, the present study integrates environmental justice theory with the activity space framework to systematically investigate differentiated pathways through which groups with varying activity ranges experience urban park green space equity and well-being. Specifically, this study aims to: (1) Develop and validate a comprehensive theoretical model that integrates socio-demographic characteristics, perceived green space quality, green space exposure, perceived social benefits, environmental justice perception, and residents’ subjective well-being; (2) Categorize the sample population into three distinct groups based on daily activity range—short-distance (local residents/short-range travelers), medium-distance (regional users/commuters), and long-distance (cross-district travelers/green space tourists)-and employ a multi-group structural equation modeling (Multi-Group SEM) approach to compare model path differences among these groups. The analysis seeks to reveal how activity space moderates the relationships between green space exposure, perceived social benefits, justice perceptions, and overall well-being. This research contributes to a micro-level understanding of the mechanisms linking urban park equity and environmental justice, offering new empirical evidence to support equitable green space planning and public health policymaking in urban contexts.

## 2 Hypothetical framework

### 2.1 Antecedents of green space exposure: socioeconomic and demographic disparities and green space quality

Urban green space exposure serves as a critical indicator for assessing the extent to which residents come into contact with and utilize green areas. It is shaped by a combination of socioeconomic and demographic factors (31). A broad consensus in the literature suggests that inequalities in green space exposure persist across income, education, and gender groups, reflecting deeper structural disparities in the distribution of green infrastructure.

At the individual level, higher levels of income, wealth, and educational attainment are associated with increased willingness and capacity to access quality environmental amenities. Consequently, middle- and high-income groups are more likely to prioritize green space access in their residential choices and daily mobility patterns (31–33). At the structural level, economic development in cities often leads to greater investment in public budgets, human resources, and land supply, all of which enhance the capacity for green space development and maintenance, thereby improving overall accessibility and usage opportunities for residents (34, 35). In terms of demographic differences, subjective perceptions of green space exposure vary across population subgroups. For instance, Sun et al. proposed a gender-based analytical framework to examine the relationship between green exposure and satisfaction. Their findings indicate that men’s satisfaction is more strongly influenced by green space accessibility indices, whereas women’s satisfaction is more affected by landscape features, suggesting divergent perceptual mechanisms between genders (36).

Green space quality is also recognized as a significant determinant of exposure. Early studies tended to operationalize quality through simplified indicators such as vegetation coverage (37, 38). Later, Gidlow et al. expanded the construct to include both natural elements (e.g., vegetation, water bodies) and built features (e.g., amenities, maintenance), emphasizing the holistic environmental condition of the green space (39). Building on this, Knobel et al. developed an 11-dimensional evaluation framework that integrates both subjective perceptions (e.g., aesthetic appeal, safety, signs of incivility) and objective attributes (e.g., land cover, biodiversity, accessibility), enabling a more comprehensive assessment of ecological function and usage potential (40).

Empirical studies highlight two primary mechanisms through which green space quality influences exposure: subjective and objective pathways. The subjective pathway emphasizes residents’ evaluations of aesthetic appeal, safety, and comfort, which in turn shape their willingness to use green spaces, thereby determining actual exposure levels (41). In contrast, the objective pathway focuses on how physical characteristics—such as canopy coverage, green space area, and amenity provision—enhance microclimatic conditions and reduce access costs, thereby increasing usage frequency. This effect is particularly pronounced in high-density urban environments (42).

Based on the above, the following hypotheses are proposed:

*H*1: Socioeconomic and demographic factors, including gender, household size, annual household income, and household financial balance, significantly influence green space exposure.

*H*2: Green space quality significantly influences green space exposure.

### 2.2 Green space quality and perceived social benefits

Perceived social benefits refer to individuals’ recognition of the positive impacts derived from social relationships and activities (43–45). Within the domain of urban green space and green infrastructure research, this concept is employed to assess residents’ evaluations of the social functions of green spaces, such as facilitating social interaction, strengthening community identity, alleviating psychological stress, and enhancing well-being and life satisfaction (46). This construct underscores the sociocultural value of green spaces and highlights the underlying mechanisms linking environmental attributes with interpersonal interactions (47–49).

Existing studies demonstrate that the quality of green environments significantly affects the perception of social benefits. On one hand, green space quality determines residents’ willingness to use these spaces, as well as the amount of time spent and frequency of activities undertaken there (50). High-quality urban green spaces typically offer safety, comfort, and aesthetic appeal, all of which enhance residents’ leisure experiences and promote physical and psychological well-being (51, 52). On the other hand, well-maintained green spaces foster place attachment and further promote mental relaxation and social interaction by offering visual enjoyment, recreational convenience, and spaces for social engagement (53–55). Thus, green space quality contributes to the enhancement of perceived social benefits through two pathways: by improving the environmental conditions for use, and by activating socio-psychological mechanisms that encourage engagement and emotional response.

Accordingly, the following hypothesis is proposed:

*H*3: Green space quality significantly influences perceived social benefits.

### 2.3 Formation of perceived green space justice: the central roles of exposure and social benefits

Urban green space justice primarily concerns the disparities among social groups in terms of accessibility, spatial distribution, and opportunities for green space use (56, 57). Early studies on environmental justice focused largely on the unequal distribution of environmental burdens such as pollution (58), whereas more recent research has expanded the scope to include spatial inequalities in green space allocation, usage, and related social processes within urban contexts (32, 59).

Empirical evidence reveals persistent deficiencies in the planning, management, and equitable distribution of green space exposure (59, 60). For example, Rao et al. used the Normalized Difference Vegetation Index (NDVI) and Green View Index (GVI) to quantify the extent and visibility of green space exposure at the activity-event level. Their study found that increased greenness enhances activity satisfaction, with a curvilinear (inverted U-shaped) relationship between green visibility and satisfaction. The results also indicated clear patterns of environmental injustice for marginalized groups (61). Similarly, Wang et al. constructed a measurement framework that captures both static and dynamic geographic contexts of green space exposure. Their findings reveal that disparities in accessibility to residential or workplace green spaces, as well as street-level greening, lead to unequal experiences of dynamic environmental exposure (62).

Beyond exposure, perceived social benefits also represent a key psychological mechanism influencing the perception of green space justice. Wolch et al. argued that individuals’ perceptions of the social functions provided by green spaces—such as safety and belonging—substantially shape access patterns among different groups, thereby affecting the realization of green space justice (57). Additional studies suggest that high-income groups are more likely to benefit from the social, recreational, and mental health advantages of high-quality green spaces, while low-income groups often face a “double disadvantage”: limited green space exposure and insufficient access to positive social experiences therein (50).

Based on this, the following hypotheses are proposed:

*H*4: Green space exposure significantly influences perceived green space justice.

*H*5: Perceived social benefits significantly influence perceived green space justice.

### 2.4 The ultimate value of perceived green space justice: a pathway to subjective well-being

Subjective well-being refers to individuals’ self-evaluation of their overall life conditions, and is commonly used to assess life satisfaction, psychological health, and general welfare (14). In recent years, urban green space has been widely recognized as an essential strategy for enhancing residents’ well-being. Numerous studies have shown that green spaces not only improve residential environments and alleviate psychological stress, but also contribute to higher levels of overall well-being (6, 63, 64). However, the acquisition of well-being is not solely determined by the quantity or size of green spaces; fairness has increasingly emerged as a crucial dimension influencing residents’ welfare outcomes (14).

Specifically, whether green spaces are equitably distributed—and whether individuals perceive their access and use as respectful and equal—constitutes a fundamental precondition for deriving psychological benefits (64). Cross-national empirical studies have indicated that while the physical size of parks does not directly affect emotional or well-being outcomes, there are significant disparities in park use experiences across different income groups (65, 66). For instance, residents of low-income communities are more likely to encounter adverse environmental conditions, such as air pollution or crime, which may reduce the psychological benefits derived from green space use (67, 68). Furthermore, under rapid urbanization, Rui et al. conducted an integrated analysis using streetscape greenery (SG), neighborhood greenery (NG), and public green space (PGS), revealing the suppressive effects of green space inequality on the well-being of socially vulnerable populations. Their findings emphasize that green space must not only exist, but also be accessible, usable, and perceptible (14). This highlights that well-being is not solely associated with the presence or quality of green spaces, but is deeply embedded in the fairness of their distribution and the psychological responses it elicits.

Accordingly, the following hypothesis is proposed:

*H*6: Perceived green space justice significantly influences subjective well-being.

**Figure 1:**
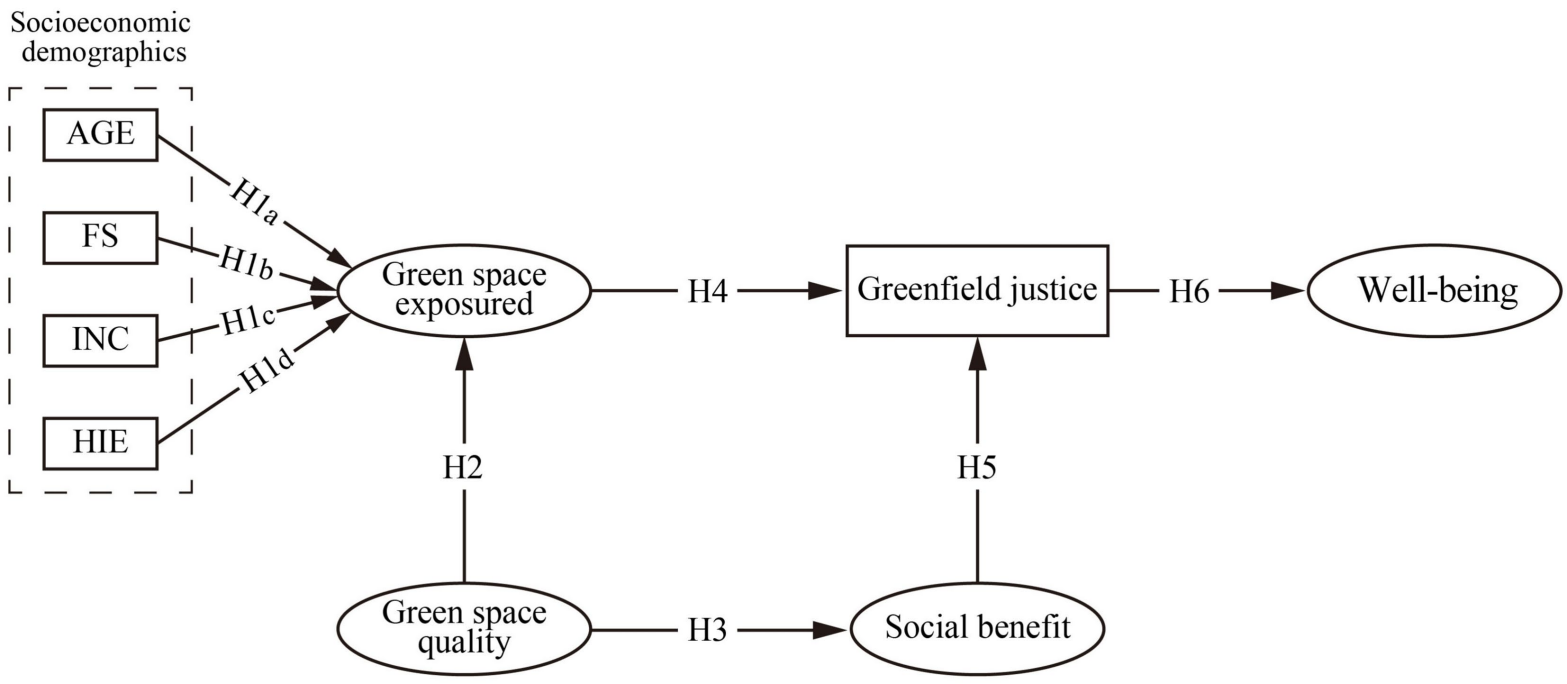
Hypothetical framework of environmental justice in urban park green spaces.

In summary, this study develops a comprehensive conceptual model to systematically examine the interrelationships among socio-demographic characteristics, green space quality, green space exposure, perceived social benefits, perceived green space justice, and subjective well-being (Figure 1).

## 3 Methodology

### 3.1 Study area

Data collection for this study was conducted in Changsha, a major city in Hunan Province, China, located between 112°53′–113°10′E longitude and 27°51′–28°41′N latitude. Changsha lies in a humid subtropical monsoon climate zone, characterized by distinct seasons and synchrony between rainfall and temperature. With abundant green space resources and a strong ecological foundation, the city provides a suitable context for urban green space research.

**Figure 2:**
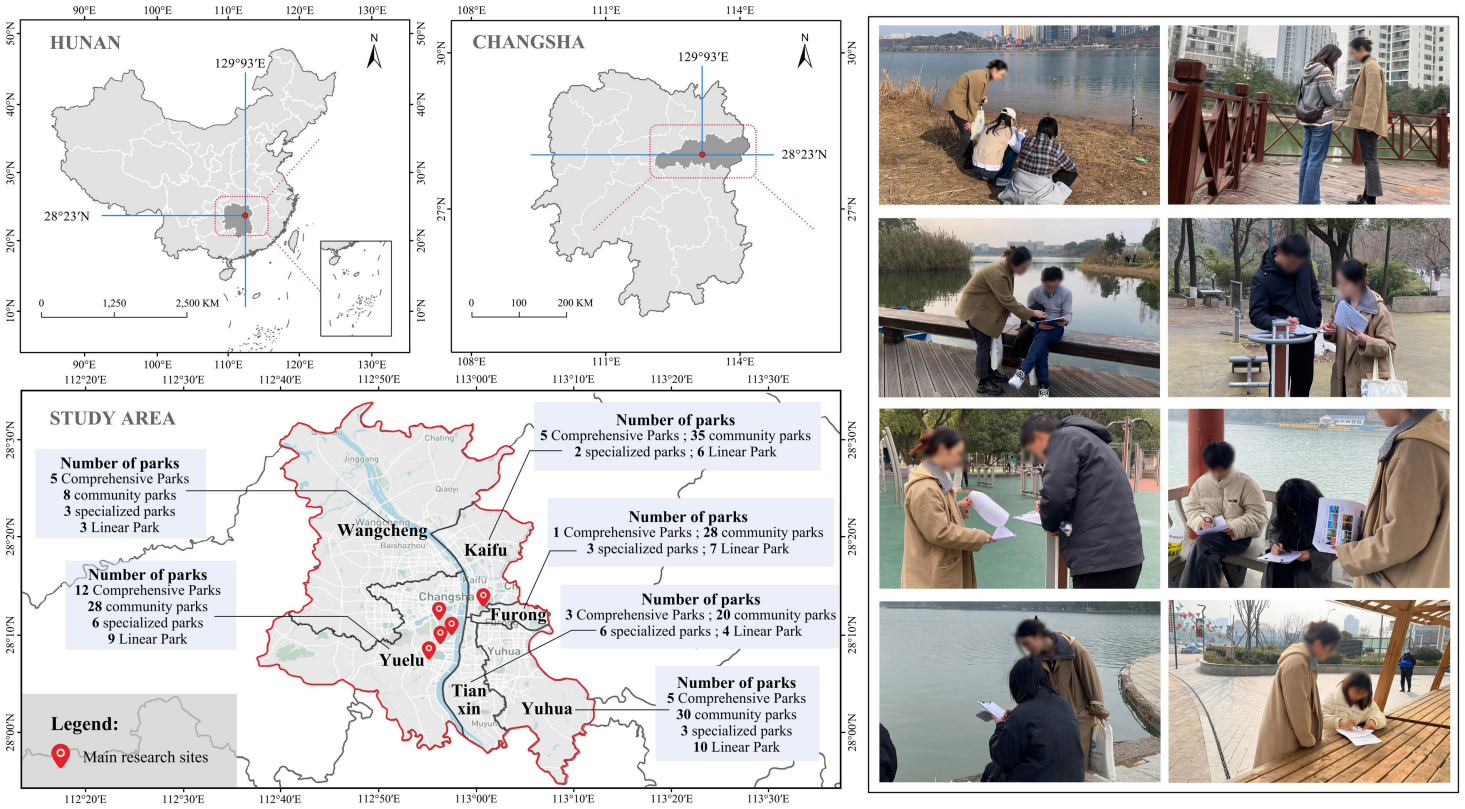
Study area, park distribution, and fieldwork photographs.

This study, based on the Changsha City Master Plan (2003–2020) (69), employed a stratified random sampling approach and ultimately selected five parks as research samples. The sample encompasses major categories of urban green spaces, including comprehensive parks, specialized parks, linear parks, and community parks. Specifically, these include Houhu Park (comprehensive park), Martyrs Park and Yuelu Mountain Scenic Area (specialized parks), Jinjiang River Greenway (linear park), and Sunshine 100 Houhai Garden (community park). The site selection comprehensively considered factors such as service population size, functional representativeness, and spatial distribution diversity, thereby ensuring that the sample adequately reflects the characteristics of Changsha’s urban green space system. Figure 2 presents the spatial distribution of the study area, a statistical summary of park types, and photographs collected during on-site field surveys.

### 3.2 Questionnaire design and data collection

This study employed a mixed-methods approach combining offline field surveys and online questionnaires via the “Wenjuanxing” platform—a widely used digital survey tool in China. Surveys were conducted simultaneously across six districts in Changsha, targeting representative urban green parks. Selection criteria for survey sites included wide service coverage and the presence of typical green space functions, ensuring the inclusion of both well-equipped parks and areas with limited green infrastructure. This approach helped avoid biased perceptions of “green justice” that may arise from sampling only high-quality parks. The selected locations represented regions with both high green space density and insufficient green provision, thereby minimizing spatial sampling bias. Data were collected from September to December 2024.

To enhance the scientific validity and contextual appropriateness of the measurement tools, a pilot survey was conducted prior to full implementation. Items with unclear logic, ambiguous wording, or contextual irrelevance were removed. During the offline survey, trained researchers provided respondents with detailed explanations of technical terms and key concepts to ensure comprehension of the questionnaire items and intent (see Figure 1). For the online version, an introductory page with visual aids and glossary definitions was presented before the survey to facilitate accurate understanding and completion. Additionally, online responses containing abnormal geolocation data or inconsistencies with local behavioral patterns were excluded to ensure spatial representativeness and alignment with the offline survey areas. After rigorous data cleaning and screening, a total of 524 valid responses were obtained, providing an adequate sample size for subsequent empirical analyses.

The final questionnaire consisted of eight sections, covering both objective behaviors and subjective perceptions: Basic demographic and behavioral variables: Age, gender, marital status, household size, type of residence, and duration of residence, were used to analyze demographic influences on green space exposure. Socioeconomic status: Education level, occupation, household income, average monthly expenditure, household financial balance, and ownership of real estate or vehicles, to assess how economic background shapes exposure. Park visit behavior and spatial attributes: Frequently visited park types, the size of the nearest neighborhood park, and the main purposes for park visits, reflecting both behavioral patterns and spatial context.

To comprehensively evaluate residents’ subjective experiences and psychological responses to urban green spaces, the questionnaire also incorporated several validated scales: Perceived Green Space Quality Scale: Adapted from the RECITAL tool (40), it includes six dimensions—species diversity, plant color, seasonal features, architectural harmony, spatial layering, and aesthetic ambiance—to capture ecological and aesthetic appeal. Perceived Social Benefits Scale (70): Measures subjective evaluations of green space functions, including mental health support, recreation, social interaction, community cohesion, quality of life, and well-being. Subjective Well-Being Scale (71): Comprises five indicators—pleasure, relaxation, vitality, sleep recovery, and enjoyment of life—to explore links between green space exposure and well-being. Perceived Green Justice: Assessed using the core question, “Do you think urban park green spaces in Changsha are fairly distributed?” to measure perceptions of fairness in green resource allocation. The use of a single item to measure green justice in this study has been empirically justified by prior research (72, 73). Green Space Exposure Scale (74, 75): Defined as the ease of access to public green space, measured through two items: “Walking time from home to the nearest park” and “Frequency of visits to the nearest park per month.” Note that walking time is reverse-coded—a higher score indicates longer distance and thus lower exposure. The construct composed of two items is also permitted in measurement practice and is commonly found in research in related fields. All items were rated using a 5-point Likert scale (1 = Strongly disagree, 2 = Disagree, 3 = Neutral, 4 = Agree, 5 = Strongly agree; Table 1).

**Table 1:** Descriptions of constructs and measurement items.

Latent construct	Item ID	Measurement item description
Perceived green space justice (SJ)	SJ	Do you think urban park green spaces in Changsha are fairly distributed?
Green space exposure (GSE)	GSE1	Walking time from your residence to the nearest park (reverse-coded).
	GSE2	Frequency of visits to the nearest park per month.
Perceived green space quality (EQ)	EQ1	Species diversity in the nearest park to your residence.
	EQ2	Plant color characteristics in the nearest park.
	EQ3	Seasonal characteristics of the nearest park.
	EQ4	Architectural harmony of the park with its surrounding environment.
	EQ5	Spatial layering of the nearest park.
	EQ6	Aesthetic ambiance and artistic impression of the park.
Perceived social benefits (SB)	SB1	Green space helps relieve anxiety and stress.
	SB2	Green space provides opportunities for recreation.
	SB3	Green space promotes contact with nature and opportunities for social interaction.
	SB4	Green space enhances community awareness and cohesion.
	SB5	Green space contributes to improving quality of life.
	SB6	Green space enhances health and subjective well-being.
Subjective well-being index (HI)	HI1	I feel happy and emotionally comfortable.
	HI2	I feel calm and relaxed.
	HI3	I feel energetic and full of vitality.
	HI4	I feel refreshed and well-rested after sleep.
	HI5	I experience a sense of enjoyment and fun in daily life.

### 3.3 Research method

#### 3.3.1 Structural equation modeling

SEM is a widely applied statistical method that enables simultaneous estimation of both the measurement relationships between observed and latent variables, and the structural path relationships among multiple latent constructs (76). Compared to traditional linear regression techniques, SEM offers the advantage of modeling multiple independent and dependent variables within a single framework. This makes it particularly suitable for examining the complex interrelationships and mechanisms among socio-demographic characteristics, perceived green space quality, green space exposure, perceived social benefits, perceived green space justice, and subjective well-being. A key strength of SEM lies in its theory-driven nature, which allows researchers to simultaneously estimate the measurement model and the structural model. This enables an integrated approach to theoretical validation and empirical testing. As a result, SEM has been extensively utilized in studies involving subjective well-being, environmental perception, and health-related behaviors (61).

The measurement model specifies how observed indicators reflect latent constructs. Its general form is expressed as Equations 1,2:

(1) $$X=\Lambda_{x}\xi +\delta$$

(2) $$Y=\Lambda_{y}\eta +\varepsilon$$

In this context, X denotes the column vector composed of observed indicators for the i -th independent latent variable, serving as the measurement variables for the latent exogenous construct ξ . Similarly, Y represents the column vector of observed indicators corresponding to the latent endogenous variable η . The matrices $\Lambda_{x}$ and $\Lambda_{y}$ are the factors loading matrices, capturing the linear relationships between the observed variables and their respective latent constructs. δ and ε denote the measurement error terms, reflecting the portions of the observed variables that are not explained by the latent variables.

The measurement model specifies how observed indicators reflect latent constructs. Its general form is expressed as Equation 3:

(3) η=Bη+Γξ+ζ

Here, η denotes the column vector of latent endogenous variables, while ξ represents the column vector of latent exogenous variables. The matrix B contains the regression coefficients among the endogenous latent variables, capturing their direct linear interrelationships. Γ is the regression coefficient matrix associated with the exogenous latent variables, reflecting the causal effects and explanatory power of the exogenous constructs on the endogenous ones. ζ represents the structural error term, accounting for the unexplained variance in the endogenous variables that is not attributable to other latent constructs within the model.

#### 3.3.2 Multi-group structural equation modeling

Building upon the baseline structural equation model, this study further adopts Multi-Group SEM to examine differences in structural path coefficients across groups with varying activity space ranges. This approach aims to explore how spatial mobility influences the mechanisms underlying green space exposure, perceived benefits, environmental justice, and subjective well-being.

Specifically, respondents were categorized into three activity space groups based on the walking distance from their residence to the nearest park: Short-distance group (500 meters–1 km): Representing local residents or short-range daily users with limited activity space; Medium-distance group (1–3 km): Including regional park users and commuters with moderate activity ranges; Long-distance group (≥3 km): Comprising individuals engaged in long-distance or cross-district travel, such as green space tourists or users with specific destination-oriented purposes. This classification enables a comparative analysis of behavioral and perceptual mechanisms associated with different spatial access levels to green space.

Multi-Group SEM allows for the simultaneous estimation of model parameters across groups and enables the testing of cross-group invariance through a stepwise constraint procedure. Key model parameters such as path coefficients, factor loadings, and error variances can be systematically evaluated for consistency or variation across the groups (77). Theoretically, this approach employs a hierarchical testing strategy—beginning with configural invariance (i.e., identical model structure), followed by increasingly stringent constraints on measurement weights, structural paths, and residual variances—to assess both the stability and heterogeneity of the model across groups (76). In this study, all Multi-Group SEM analyses were conducted using the built-in multi-group analysis module in AMOS.

## 4 Results

### 4.1 Sample characteristics

Table 2 presents the demographic profile of the 524 respondents. The age distribution is primarily concentrated in the 41–54 age group (29.0%), followed by 18–23 (18.9%), 31–40 (16.8%), and 55–64 years (16.4%). Respondents under 18 or over 65 years of age accounted for a relatively small proportion. In terms of gender, females constituted a slightly higher percentage (58.4%) than males (41.6%). Regarding marital status, the majority were married (60.7%), followed by unmarried individuals (34.0%) and divorced respondents (5.3%). Household size was predominantly composed of three-member (32.2%) and four-member (29.0%) families, with larger households (five or more members) making up 23.5%. In terms of housing type, group-owners (purchased housing with family or roommates) accounted for 35.9%, followed by solo homeowners (25.6%), and dormitory residents (22.5%). Regarding length of residence, over half of respondents (56.9%) had lived in their current residence for more than 5 years, while 18.1% reported living there for less than 1 year, indicating relatively stable residential patterns.

**Table 2:** Basic demographic characteristics of respondents.

Variable	Category	Frequency	Percentage (%)
Age	Under 18	13	2.5
	18–23	99	18.9
	24–30	79	15.1
	31–40	88	16.8
	41–54	152	29
	55–64	86	16.4
	65 and above	7	1.3
Gender	Male	218	41.6
	Female	306	58.4
Marital status	Unmarried	178	34
	Married	318	60.7
	Divorced	28	5.3
Household size	1 person	26	5
	2 persons	54	10.3
	3 persons	169	32.2
	4 persons	152	29
	5 or more people	123	23.5
Type of residence	Rented alone	60	11.4
	Shared rental	24	4.6
	Dormitory (school or workplace)	118	22.5
	Owned, living alone	134	25.6
	Owned, living with family/others	188	35.9
Length of residence	≤ 6 months	40	7.6
	6 months – 1 year	55	10.5
	1–2 years	39	7.4
	2–3 years	37	7.1
	3–4 years	29	5.5
	4–5 years	26	5
	More than 5 years	298	56.9

Table 3 outlines the socioeconomic characteristics of respondents. The sample shows diverse occupational backgrounds, with students comprising the largest group (26.0%), followed by farmers, workers, and employees (21.8%). Education levels were relatively high, with 33.8% holding a bachelor’s degree and 9.4% holding a master’s degree or above. Respondents with a high school or vocational diploma made up 25.4%. Regarding annual household income, the majority fell below CNY 100,000 (Chinese Yuan), with 29.6% earning less than CNY 50,000 and 26.3% earning between CNY 50,000 and 100,000. High-income households (above CNY 300,000) represented less than 10%. Monthly expenditures were mostly concentrated in the CNY 2,000–10,000 range, with 34.9% spending CNY 2,000–5,000 and 29.2% spending CNY 5,000–10,000. High-expenditure households (over CNY 15,000 per month) accounted for 9.9%. In terms of household financial status, 36.6% reported a “balanced income and expenditure,” while 14.7% experienced “occasional surplus,” and 10.9% reported “consistent surplus.” Conversely, 17.4% indicated it was “difficult to balance income and expenditure,” and 20.4% reported it was “occasionally difficult.” Most respondents owned one residential property (46.8%), while 30.3% owned two properties; only 11.1% were non-homeowners. Over half of the respondents reported owning one car, with 22.7% owning two cars; non-car owners accounted for 21.0%.

**Table 3:** Socioeconomic characteristics of respondents.

Variable	Category	Frequency	Percentage (%)
Occupation	Unemployed	40	7.6
	Student	136	26
	Retired	35	6.7
	Farmers, workers, employees	114	21.8
	Business executives	30	5.7
	Professionals	23	4.4
	Government staff	39	7.4
	Other	107	20.4
Education level	Middle school or below	93	17.7
	High school or vocational school	133	25.4
	Junior college (3-year diploma)	72	13.7
	Bachelor’s degree	177	33.8
	Master’s degree or above	49	9.4
Annual household income (CNY)	< 50,000	155	29.6
	50,000–100,000	138	26.3
	100,000–150,000	91	17.4
	150,000–200,000	57	10.9
	200,000–300,000	28	5.3
	300,000–400,000	19	3.6
	400,000–500,000	15	2.9
	> 500,000	21	4
Monthly household expenditure (CNY)	< 2,000	94	17.9
	2,000–5,000	183	34.9
	5,000–10,000	153	29.2
	10,000–15,000	42	8
	> 15,000	52	9.9
Household financial balance	Difficult to balance	91	17.4
	Occasionally difficult	107	20.4
	Balanced	192	36.6
	Occasional surplus	77	14.7
	Surplus	57	10.9
Home ownership	No property	58	11.1
	1 property	245	46.8
	2 properties	159	30.3
	3 properties	32	6.1
	4 or more properties	30	5.7
Car ownership	No vehicle	110	21
	1 vehicle	276	52.7
	2 vehicles	119	22.7
	3 vehicles	14	2.7
	4 vehicles	5	1

**Figure 3:**
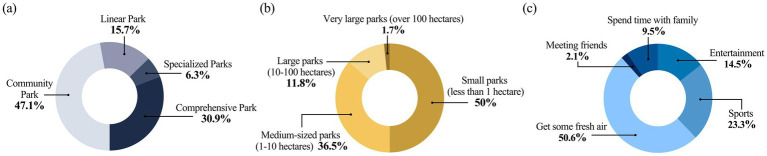
Respondents’ park visitation behaviors and spatial attributes: **(a)** primary types of parks visited; **(b)** size of the nearest neighborhood park; **(c)** primary purposes for visiting urban parks.

Figure 3 illustrates respondents’ park visitation behaviors and the spatial attributes of green space use. Figure 3a shows that community parks were the most frequently visited (47.1%), followed by comprehensive parks (30.9%) and linear parks (15.7%), with specialized parks accounting for only 6.3%. Figure 3b presents the size of the nearest parks: small parks (less than 1 hectare) were most common (50.0%), followed by medium-sized parks (1–10 hectares, 36.5%), while large and extra-large parks were less common (11.8 and 1.7%, respectively). Figure 3c displays park visit motivations. Over half of respondents (50.6%) visited parks primarily to enjoy nature and breathe fresh air. Others cited exercise (23.3%) and recreation (14.5%) as main reasons, while smaller percentages visited for family time (9.5%) and socializing with friends (2.1%).

In addition, respondents were divided into three groups based on their daily activity space range, defined by walking distance from home to the nearest park: Short-distance group (500 m–1 km): 158 individuals (30.2%). Medium-distance group (1–3 km): 215 individuals (41.0%). Long-distance group (≥3 km): 151 individuals (28.8%). This classification was used for subsequent Multi-Group SEM analyses.

### 4.2 Structural equation modeling results

#### 4.2.1 Reliability and validity analysis

The reliability and validity of the measurement scales were examined through internal consistency testing and factor analysis. First, Cronbach’s alpha coefficients were calculated for each latent construct. The results indicated high internal reliability across all core constructs: Subjective Well-Being (*α* = 0.961), Perceived Green Space Quality (α = 0.852), Perceived Social Benefits (α = 0.920), and Green Space Exposure Frequency (α = 0.866). All values exceeded the commonly accepted threshold of 0.80, suggesting strong internal consistency.

To assess structural validity, the Kaiser–Meyer–Olkin (KMO) test and Exploratory Factor Analysis (EFA) were conducted. The KMO values were as follows: Subjective Well-Being = 0.900, Perceived Green Space Quality = 0.887, Perceived Social Benefits = 0.896, and Green Space Exposure = 0.500. According to established statistical guidelines (78–80), a KMO value of 0.50 is considered the minimum acceptable level for factor analysis. Thus, although the KMO for Green Space Exposure is at the lower bound, it still meets the criterion for factorability. The relatively modest value primarily reflects the fact that this construct was measured with only two items, one of which was reverse-coded. Nonetheless, constructs measured with one or two items are not uncommon in environmental psychology and environmental justice research (72, 73, 81, 82). These findings support the reliability and construct validity of the measurement instruments, thereby justifying their use in subsequent structural modeling and hypothesis testing.

#### 4.2.2 Structural model fit results

SEM was conducted to estimate and evaluate the proposed theoretical framework. After necessary model modifications and re-specification, the final model exhibited acceptable fit statistics. As shown in Table 4, key fit indices were used to assess model adequacy, including: Chi-square (χ^2^); Root Mean Square Error of Approximation (RMSEA); Comparative Fit Index (CFI); Tucker–Lewis Index (TLI); Chi-square/degrees of freedom ratio (χ^2^/df). According to conventional SEM evaluation standards, a good model fit is indicated by: χ^2^/df ≤ 3; RMSEA ≤ 0.08; CFI ≥ 0.90; TLI ≥ 0.90 (83–85). As shown in Table 4, all indices fall within acceptable ranges, indicating that the final model demonstrates a satisfactory overall fit to the observed data.

**Table 4:** Model fit indices for the final structural equation model.

CMIN	CMIN/DF	IFI	CFI	RMSEA
809.156	2.819	0.933	0.933	0.059

#### 4.2.3 Path coefficients and hypothesis testing

**Figure 4:**
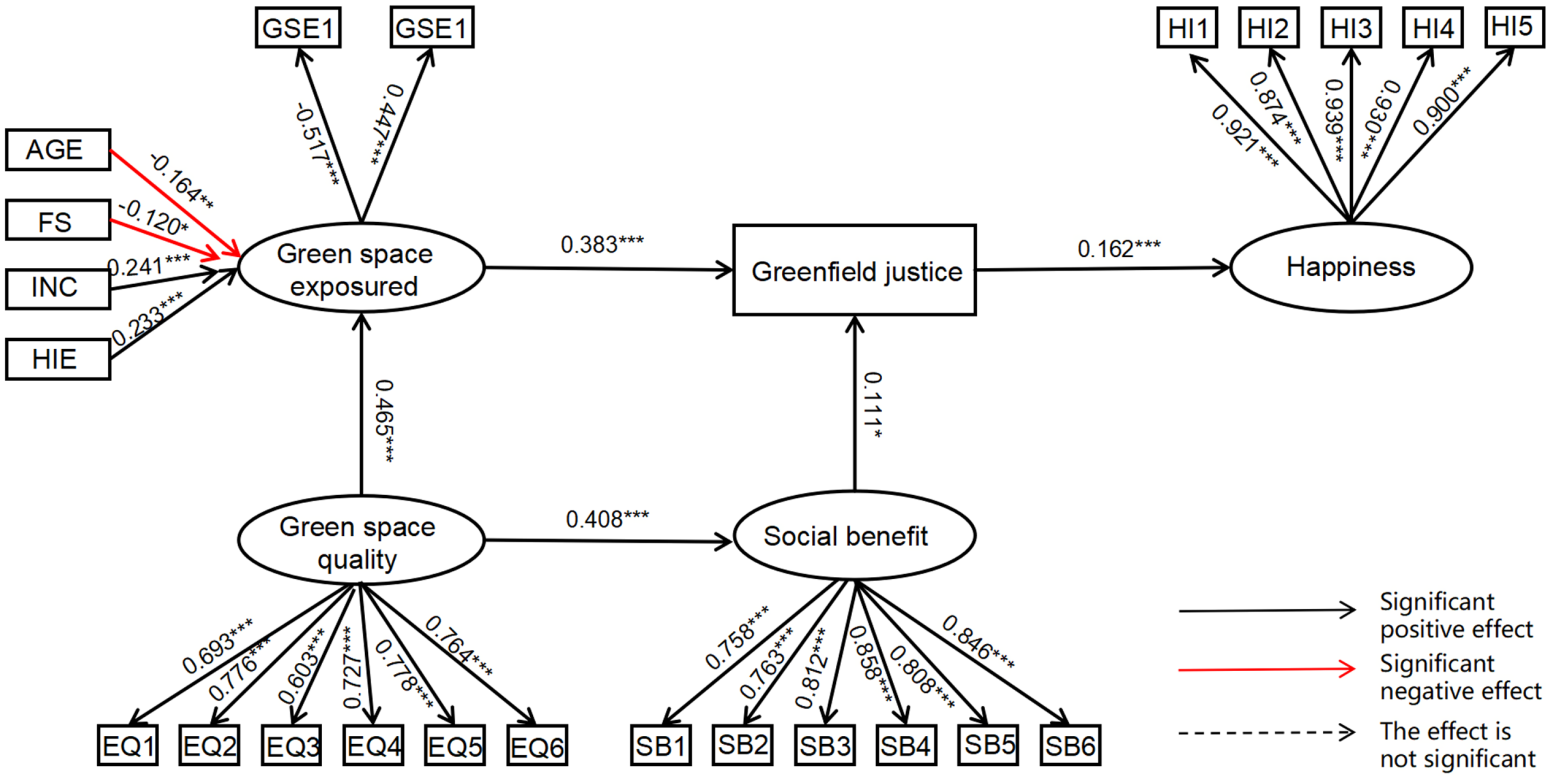
Results of structural equation modeling. *0.01 < P ≤ 0.05; **0.001 < P ≤ 0.01; ***P ≤ 0.001.

The structural equation modeling results (Figure 4; Table 5) indicate that gender has a significant negative effect on green space exposure (*β* = −0.164, *p* = 0.008), and household size also shows a significant negative relationship with green space exposure (*β* = −0.120, *p* = 0.049). In contrast, annual household income (*β* = 0.241, *p* < 0.001) and household income-expenditure balance (*β* = 0.233, *p* = 0.001) both exhibit significant positive effects on green space exposure. In addition, green space quality significantly influences both green space exposure (*β* = 0.465, *p* < 0.001) and perceived social benefits (*β* = 0.408, *p* < 0.001). Green space exposure has a significant positive effect on perceived green justice (*β* = 0.383, *p* < 0.001), and perceived social benefits also have a significant positive effect on green justice perception (*β* = 0.111, *p* = 0.014). Furthermore, perceived green justice has a significant and positive impact on subjective well-being (*β* = 0.162, *p* < 0.001).

**Table 5:** Structural model results.

Path Relationship	Estimate	*p*-value
Gender → Green Space Exposure	−0.164	0.008
Household Size → Green Space Exposure	−0.120	0.049
Annual Household Income → Green Space Exposure	0.241	***
Income–Expenditure Balance → Green Space Exposure	0.233	0.001
Green Space Quality → Green Space Exposure	0.465	***
Green Space Quality → Social Benefits	0.408	***
Green Space Exposure → Perceived Green Justice	0.383	***
Social Benefits → Green Justice	0.111	0.014
Green Justice → Well-being	0.162	***

*** indicates *p* < 0.001; ** indicates *p <* 0.01; * indicates *p* < 0.05.

The measurement model results (Table 6) indicate that all observed variables for the latent constructs demonstrate good convergent validity, with all factor loadings reaching statistically significant levels (*p* < 0.001) and generally high standardized coefficients. Notably, GSE1 is a reverse-coded item, where a higher score represents a longer distance to the nearest park and thus a lower level of green space exposure. Therefore, its negative factor loading is consistent with theoretical expectations.

**Table 6:** Measurement model results.

Latent variable		Estimate	*p*-value
Well-being	HI1	0.921	***
	HI2	0.874	***
	HI3	0.939	***
	HI4	0.930	***
	HI5	0.900	***
Green space quality	EQ1	0.693	***
	EQ2	0.776	***
	EQ3	0.603	***
	EQ4	0.727	***
	EQ5	0.778	***
	EQ6	0.764	***
Social benefits	SB1	0.758	***
	SB2	0.763	***
	SB3	0.812	***
	SB4	0.858	***
	SB5	0.808	***
	SB6	0.846	***
Green space exposure	GSE1	−0.517	***
	GSE2	0.447	***

*** indicates *p* < 0.001; ** indicates *p <* 0.01; * indicates *p* < 0.05.

#### 4.2.4 Multi-group analysis

As shown in Table 7, the multi-group structural equation models exhibited good fit under all constraint conditions. The unconstrained model demonstrated satisfactory fit indices (CFI = 0.928, RMSEA = 0.036), and the introduction of constraints on measurement weights, structural weights, and structural residuals resulted in only minimal changes in model fit (CFI ≥ 0.928; RMSEA ≤ 0.035), with all CMIN/DF values below 2—meeting the criteria for good model fit. Although a slight decline in fit was observed in the stage of measurement residual constraints (CFI = 0.903, RMSEA = 0.039), these indices remained within acceptable thresholds. Overall, all models satisfied standard fit criteria (CMIN/DF < 3, IFI/CFI > 0.90, RMSEA < 0.08), indicating good model performance across the three distance-based groups (near, medium, and far), thereby supporting subsequent cross-group comparisons.

**Table 7:** Model fit indices for multi-group analysis.

Levels of invariance testing	CMIN	DF	*p*	CMIN/DF	IFI	CFI	RMSEA
Unconstrained	1407.815	843.000	0.000	1.670	0.930	0.928	0.036
Measurement weights	1443.730	877.000	0.000	1.646	0.929	0.928	0.035
Structural weights	1448.426	891.000	0.000	1.626	0.930	0.929	0.035
Structural residuals	1502.725	925.000	0.000	1.625	0.927	0.927	0.035
Measurement residuals	1744.985	983.000	0.000	1.775	0.903	0.903	0.039

The invariance test results (Table 8) further confirmed that the model remained invariant across groups in terms of measurement weights (*p* = 0.379), structural weights (*p* = 0.767), and structural residuals (*p* = 0.156), with changes in comparative fit index (ΔCFI) remaining minimal (all ≤ 0.001), suggesting equivalence in measurement and structural paths across the three groups. However, in the stage of measurement residuals, the test showed statistical significance (*p* = 0.000), and the ΔCFI dropped to −0.025, indicating the presence of group-specific measurement errors. Nonetheless, the structural paths demonstrated overall stability across groups.

**Table 8:** Invariance test results for multi-group SEM.

Levels of invariance testing	ΔCMIN	ΔDF	*p*	ΔCMIN/DF	ΔIFI	ΔCFI	ΔRMSEA
Measurement weights	35.915	34.000	0.379	−0.024	−0.001	0.000	−0.001
Structural weights	40.611	48.000	0.767	−0.044	0.000	0.001	−0.001
Structural residuals	94.910	82.000	0.156	−0.045	−0.003	−0.001	−0.001
Measurement residuals	337.170	140.000	0.000	0.105	−0.027	−0.025	0.003

**Figure 5:**
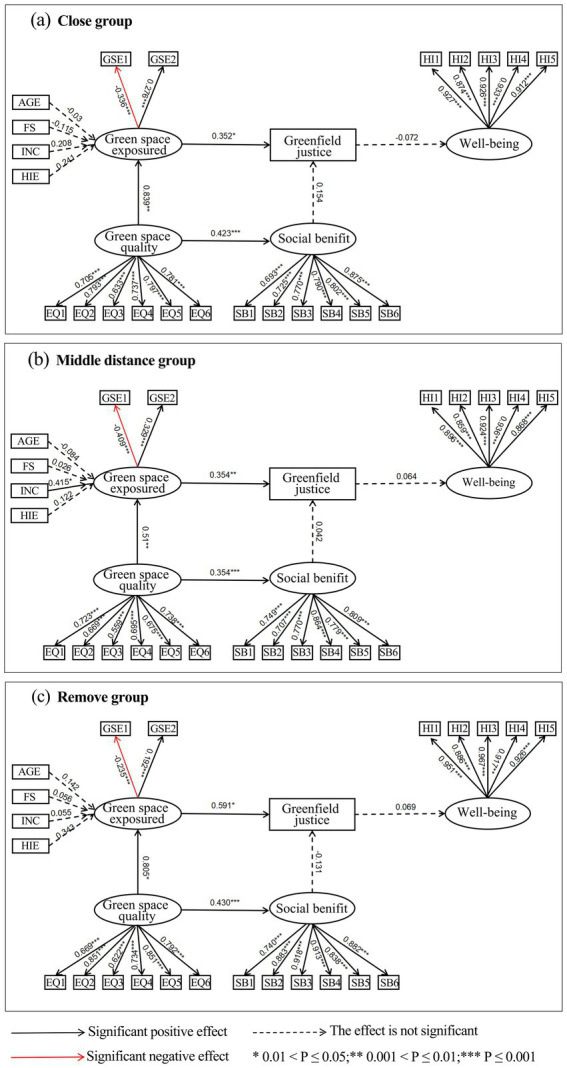
Multi-group SEM results by distance group. *0.05 < p ≤ 0.10; **0.01 < p ≤ 0.5; ***p ≤ 0.01. **(a)** Short-distance group (500 m–1 km), **(b)** Medium-distance group (1–3 km), **(c)** Long-distance group (≥3 km).

Figure 5 presents the structural and measurement model results across the three distance-based groups. The measurement indicators for subjective well-being, perceived green space quality, perceived social benefits, and green space exposure were all statistically significant, confirming adequate measurement consistency. Among the near-distance group (500 m–1 km), green space quality exerted a significant positive effect on green space exposure (*β* = 0.839, *p* = 0.003), and also significantly influenced perceived social benefits (*β* = 0.423, *p* < 0.001). Furthermore, green space exposure significantly affected perceived green justice (*β* = 0.352, *p* = 0.013), whereas the effect of perceived social benefits on green justice was not statistically significant (*p* = 0.075), nor was the impact of perceived green justice on well-being.

In the medium-distance group (1 km–3 km), annual household income showed a significant positive association with green space exposure (*β* = 0.415, *p* = 0.011). Green space quality significantly influenced both green space exposure (*β* = 0.510, *p* = 0.004) and perceived social benefits (*β* = 0.354, *p* < 0.001), while green space exposure had a significant effect on perceived green justice (*β* = 0.354, *p* = 0.008). However, no other paths in this group reached statistical significance.

For the far-distance group (≥ 3 km), green space quality had significant positive effects on both green space exposure (*β* = 0.805, *p* = 0.042) and perceived social benefits (*β* = 0.430, *p* < 0.001). Green space exposure significantly influenced perceived green justice (*β* = 0.591, *p* = 0.044). Nevertheless, neither the path from perceived social benefits to green justice nor the path from green justice to well-being reached statistical significance in this group.

## 5 Discussion

### 5.1 Path mechanism from urban green justice to well-being

This study, grounded in environmental justice theory and the activity space perspective, systematically investigates the formation mechanism of urban park green justice, with particular attention to differences in perceptions among groups with varying activity ranges. The findings largely support the research hypotheses: the environmental quality of urban park green spaces not only significantly affects residents’ green space exposure and perceived social benefits but also enhances their well-being through the perception of green justice. This suggests that high-quality green environments serve as a prerequisite for attracting public use and fostering positive social experiences, while perceived green justice plays a potential mediating role between environmental conditions and mental health outcomes.

Furthermore, the study finds that both annual household income and household income–expenditure balance have significant positive effects on green space exposure. This indicates that groups with stronger economic conditions are more likely to increase their opportunities for green space access. This result is consistent with the findings of Yang et al., who, based on spatial justice, social stratification theory, and Rawls’ theory of justice, argue that structural disparities exist in access to urban green spaces and the associated health benefits across socioeconomic groups. In this structure, high-SES groups enjoy significantly better opportunities for accessing green spaces and thus gain more health-related advantages (75). This inequality highlights the necessity for urban green space planning and allocation to place greater emphasis on the equity demands and practical accessibility of socially disadvantaged groups, thereby ensuring the fair effectiveness of environmental resources in promoting mental well-being.

### 5.2 Spatial variations in green justice under activity space ranges

Further multi-group analysis reveals that activity space plays a significant structural role in the mechanism of urban green justice. First, across all three distance-based groups, the path from “green space exposure → perceived green justice” remains significantly positive, indicating that higher levels of green space exposure are more likely to elicit stronger perceptions of fairness in the distribution of urban green spaces. In other words, greater exposure to green space can stimulate individuals’ subjective evaluation of justice.

Second, the paths from “green space quality to green space exposure” and “green space quality to perceived social benefits” are also significant in all groups. This suggests that regardless of differences in spatial location, high-quality green spaces consistently promote both the frequency of green space use and residents’ recognition of its social benefits. These three paths form a stable structural mechanism in the relationship between green justice and well-being, demonstrating strong cross-group consistency.

However, beyond these three core paths, the remaining path relationships do not reach statistical significance across the groups. This may reflect that under different activity space conditions, the relationships between certain psychological perceptions and well-being are not yet robust, possibly constrained by daily usage habits, levels of participation in social activities, and variations in park functions. For instance, within the short-distance group, community parks have become an integral part of daily life, and their social functions may have been “internalized,” thus no longer significantly influencing perceptions of justice. This suggests that traditional static models may underestimate the mechanisms of “functional saturation” and “diminishing marginal effects” in everyday green space use.

In the long-distance group, green space usage is more “occasional” and “purpose-driven,” typically involving infrequent “green tourism” focused on landscape experiences and recreational functions. This group pays less attention to fairness, resulting in a non-significant path from perceived green justice to well-being, reflecting a disconnect between a “consumption-oriented” usage model and a “belonging-oriented” path to Well-being.

In addition, within the mid-distance group, a significant positive path from “household annual income to green space exposure” is observed, which is not present in the other two groups. This indicates that the green space usage behavior of the mid-distance group is more strongly influenced by economic conditions. This may reflect that accessing green spaces at this range often requires more reliance on transportation or other activity resources, and economically better-off individuals are more capable of overcoming spatial barriers, thereby increasing their level of green space exposure.

### 5.3 Implications

At the practical level, the research findings provide strategic guidance for the differentiated design of urban green spaces. First, the descriptive analysis indicates that small community parks and “enjoying nature” are the main usage scenarios at present. Therefore, priority should be given to enhancing the ecological and landscape quality of community parks to improve residents’ sense of access to daily leisure and natural experiences. Second, the measurement model further demonstrates that landscape elements such as plant color and spatial layering are particularly critical to residents’ experiences. Future green space design should focus on optimizing these visual and spatial features, for example, by incorporating plant combinations with seasonal variation, enhancing visual coherence and spatial layering, and creating multifunctional, walkable spatial structures to increase visual appeal and spatial engagement.

Targeted strategies should be adopted for different activity range groups. For the short-distance group, which mainly focuses on spatial layering and aesthetic ambiance, it is recommended to enhance spatial diversity and layering in community parks, such as introducing undulating terrain, small water features, shaded walkways, and quiet resting areas, to create spaces suitable for exploration and contemplation that fulfill the needs for frequent, short-term, deep contact with nature. The mid-distance group pays more attention to aesthetic ambiance and species diversity, while also considering accessibility and economic affordability. For this group, regional parks should be enriched with diverse plant communities, ecological education facilities, and trail networks, while improving public transportation and pedestrian connections to enhance accessibility and reduce travel barriers. The long-distance group is particularly concerned with plant color and spatial layering and tends to use parks primarily for leisure and visual enjoyment. Therefore, large urban or suburban parks should be designed with large-scale, colorful, and open landscapes and equipped with facilities suitable for families and group activities to meet the needs of recreational users or short-distance tourists.

It is worth noting that this study categorizes activity range groups based on self-reported travel distance and usage behaviors of respondents, focusing on the concept of “functional distance in everyday use” rather than fixed divisions based solely on geometric distance. Thus, the same park may indeed present different spatial relationships for different groups. The core purpose of this research is to identify differences in individuals’ actual usage and perceptions in real-life contexts. In terms of design and policy, this concept of “relative distance” emphasizes the need to pay closer attention to residents’ actual travel patterns, activity trajectories, and psychological perceptions. Urban green space planning should comprehensively consider walkability, transportation accessibility, and functional zoning, promoting a transformation from single-use “neighborhood green spaces” to “multifunctional, multi-layered green space networks” that can meet the diverse needs of different population groups.

At the theoretical level, this study expands the perspective of environmental justice theory and green equity research. It is the first to systematically integrate the complex pathways between green space quality, green space exposure, perceived justice, perceived social benefits, and well-being, addressing the limitations of previous studies that focused on single pathways. Furthermore, by introducing groupings based on spatial distance, this study reveals group-based differences in residents’ perceptions and usage of green space, offering important theoretical innovations.

### 5.4 Limitations

Despite providing systematic evidence, this study has several limitations. First, the data were primarily collected in Changsha, which introduces a certain degree of regional limitation. Future research could be extended to different cities or cross-national comparisons to test the broader applicability of the model. Second, the study is based on cross-sectional survey data. Although it reveals the relational pathways among urban green space quality, green space exposure, perceived justice, and subjective well-being, it does not allow for direct causal inference and cannot fully capture the dynamic evolution and spatiotemporal complexity of green equity. Future studies could incorporate longitudinal tracking, experimental designs, or natural experiments to further verify the causal direction and temporal features of these pathways. Third, although random sampling was employed at the selected sites, we acknowledge that some vulnerable groups may still be underrepresented. Future research is therefore recommended to adopt strategies such as stratified sampling to enhance the inclusiveness and representativeness of the sample. In addition, as green space exposure was measured using only two items, its convergent validity proved insufficient. Future studies may improve its reliability and validity by increasing the number of items, refining the scale structure, and expanding the sample size. Finally, this study relies on self-reported data and questionnaire surveys, mainly focusing on subjective perceptions and behavioral characteristics, while lacking detailed measurements of objective behavioral trajectories and dynamic spatial exposure. Future research could introduce emerging technologies such as mobile signaling data or spatiotemporal activity tracking, integrating dynamic activity spaces with multi-source data to reconstruct spatial justice models. This would allow for a more precise exploration of the complex mechanisms underlying green equity.

## 6 Conclusion

This study, grounded in environmental justice theory and the activity space perspective, constructed and empirically tested a structural equation model integrating green space quality, green space exposure, perceived social benefits, perceived green justice, and subjective well-being, based on Changsha residents’ perceptions of urban park green spaces. Furthermore, a multi-group comparison was conducted to reveal the differentiated mechanisms of green justice across groups with varying activity ranges (near/mid/far distance). The main conclusions are as follows:

1. The quality of urban park green spaces significantly affects both green space exposure and perceived social benefits and further enhances subjective well-being through the mediation of perceived green justice.
2. The three structural paths, “Green Space Quality to Green Space Exposure,” “Green Space Quality to Perceived Social Benefits,” and “Green Space Exposure to Perceived Green Justice”—remain significantly positive regardless of activity range. These constitute relatively stable mechanisms influencing green justice and well-being in urban green spaces.
3. Only in the mid-distance group does “Household Income → Green Space Exposure” show a significant positive effect, suggesting that for this population, access to transportation and economic capacity are critical conditions influencing green space accessibility.
4. Different groups exhibit distinct preferences regarding green space quality elements: the near-distance group values spatial esthetics and a sense of atmosphere; the mid-distance group emphasizes biodiversity and transportation convenience; and the far-distance group prioritizes large-scale visual impact and recreational facilities.

This study extends the application scope of environmental justice theory, offering both theoretical support and empirical evidence for understanding how perceived fairness in urban green space distribution influences subjective well-being. It also provides practical guidance for equity-oriented and functionally targeted urban green space planning.

## Data Availability

The raw data supporting the conclusions of this article will be made available by the authors, without undue reservation.
